# Supplementary material for: Diagnosing Acute Cellular Rejection after Paediatric Liver Transplantation—Is There Room for Interleukin Profiles?
Source: Children (Basel). 2023 Jan 7;10(1):128. doi: 10.3390/children10010128 (PMC9857115; doi:10.3390/children10010128)
Supplement: Supplementary file 1 [file children-10-00128-s001.zip › children-2022589-SM.pdf]

Supplementary Figure S1: Number of biopsie per time interval after Plt.

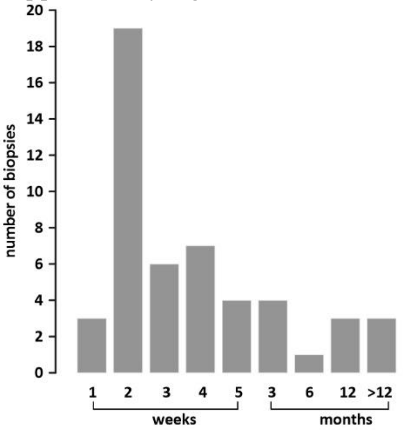

Supplementary Table S1: soluble cytokine levels in rejectors and non-rejectors.

**cytokines**		**synonym**	**rejection** mean ± SD	**ro rejection** mean ± SD	**T (df)**	**p**	**Effect size (Cohen's d)**
TH1 responses	IFN-γ		2.4±2.0	2.1±1.7	0.6 (50)	0.56	
	IL-2		1.5±1.5	1.4±1.2	0.2 (50)	0.80	
	IL-12(p70)		1.8±1.6	1.5±1.0	0.6 (50)	0.52	
	G-CSF		4.3±0.7	4.2±0.5	0.8 (50)	0.40	
	GM-CSF		1.4±1.2	1.4±1.1	0.1 (50)	0.96	
	TNF-α		2.9±0.8	2.7±0.6	1.0 (50)	0.30	
TH2 responses	IL-4		0.6±0.6	0.6±0.5	0.5 (50)	0.60	
	IL-5		2.4±1.0	2.8±0.6	-1.4 (50)	0.16	
	IL-10		2.2±1.1	1.7±1.1	1.6 (50)	0.13	
	IL-13		0.8±1.2	0.8±1.3	0.1 (50)	0.9	
TH9 responses	IL-9		3.6±0.6	3.4±0.3	1.1 (50)	0.27	
TH17 responses	IL-17		2.2±1.5	1.6±1.4	1.3 (50)	0.21	
	IL12p40	IL-23	5.5±1.1	5.1±1.1	1.4 (51)	0.18	
polyfunctional	IL-1α		-0.7±1.5	-1.0±1.7	0.6 (48)	0.57	
	IL-1β		0.8 ±0.9	0.5±0.8	1.3 (50)	0.20	
	IL-1RA		5.2±0.9	5.1±0.8	0.3 (50)	0.79	
	IL-3		5.9±0.7	5.5±0.8	1.7 (51)	0.09	0.8
	IL-6		2.5±1.1	2.0±1.0	1.8 (50)	0.08	1.1
	IL-7		1.1±1.2	1.5±0.7	-1.5 (50)	0.15	
	IL15		1.5±1.5	1.8±1.6	-0.7 (50)	0.49	
	IL-16		6.3±0.8	5.9±0.7	2.1 (51)	**0.04**	1.1
	IL-18		5.0±1.1	4.2±1.2	2.6 (51)	**0.01**	1.1
	IFN- α2		4.1±0.5	3.9±0.4	1.6 (51)	**0.11**	
	LIF		3.7±0.6	3.6±0.6	0.6 (51)	0.53	
angiogenic factor	VEGF		3.4±1.1	2.8±0.9	1.6 (50)	0.11	
chemokines		**synonym**					
CCL chemokines	CCL2	MCP-1	2.7±1.2	2.5±0.8	0.4 (50)	0.68	
	CCL3	MIP-1	1.1±1.1	0.7±0.8	1.3 (50)	0.20	
	CCL4	MIP-1	4.1±0.6	3.8±0.4	2.2 (50)	0.03	0.5
	CCL5	RANTES	7.5±0.8	7.5±0.7	-0.1 (50)	0.99	
	CCL7	MCP-3	4.5±0.7	4.3±1.2	0.7 (20.2)	0.52	
	CCL11	Eotaxin	3.7±0.7	3.4±0.6	1.8 (50)	0.07	0.7
	CCL27	CTACK	5.8±0.9	6.0±0.5	-1.0 (49)	0.33	
CXCL chemokines	CXCL1	Gro-a	4.4±0.7	4.4±0.5	0.4 (51)	0.68	
	CXCL8	IL-8	3.1±1.4	2.2±0.8	2.4 (50)	0.02	1.2

	CXCL9	MIG	7.0±1.0	6.0±1.7	2.4 (22)	0.02	1.3
	CXCL10	IP-10	6.4±1.2	5.6±1.1	2.2 (50)	0.04	1.2
	CXCL12	SDF-1 α	5.9±0.6	5.8±1.7	0.2 (18)	0.82	
**growth factors**	M-CSF		3.0±1.0	2.6±2.1	0.7 (19.8)	0.50	
	SCF		4.5±0.8	4.7±2.0	-0.3 (18)	0.74	
	SCGF- β		10.0±0.7	9.4±1.0	2.5 (51)	0.01	0.84
	PDGF		5.4±1.5	5.3±1.3	0.2 (50)	0.86	
	HGF		6.4±1.3	5.6±1.1	2.2 (51)	0.04	0.52
	FGF β		2.7±0.7	2.6±0.7	0.5 (50)	0.60	
	MIF		8.2±1.2	7.4±1.0	2.4 (51)	0.02	1.1
	TNF- β	LT α	1.9±0.9	1.8±1.6	0.4 (51)	0.69	
soluble surface molecules	sCD25	IL-2Rα	6.3±1.4	5.5±1.5	1.7 (51)	0.09	1.4
	ICAM-1		10.9±0.5	9.8±2.3	1.9 (16.8)	**0.07**	1.4
	VCAM		10.2±0.8	10.0±0.7	1.3 (51)	0.19	
	TRAIL		4.7±0.9	4.9±2.2	-0.5 (18.5)	0.63	

Supplementary Table S2: Differences in cytokine levels between rejectors and non-rejectors based on timing of rejection.

**< 20 days**<br>**rejection n=19**<br>**no rejection n=6**	rejection<br>Ln (conc) [pg/ml] mean±SD	no rejection<br>Ln (conc) [pg/ml] mean±SD	T (df)	P	Effect size<br>Cohen's d
IL-1b	1.1±0.7	0.3±0.7	2.5 (23)	0.02	0.7
IL-4	0.7±0.3	0.4±0.3	2,3 (23)	0.01	0.3
CXCL8	3.6±1.5	2.5±0.5	2.6 (22.9)	0.02	1.4
CCL11	4.0±0.6	3.2±0.5	2.9 (23)	0.01	0.6
IL-18	5.2±1.2	3.9±0.9	2.5 (24)	0.02	1.1
HGF	6.5±1.4	5.3±0.6	2.1 (24)	0.046	1.3
M-CSF	3.0±1.1	1.6±0.9	2.8 (24)	0.01	1.1
CXCL12	5.8±0.7	6.8±0.8	-2.9 (24)	0.01	0.7
TNFβ	2.1±0.8	1.2±1.0	2.2 (24)	0.04	0.9
**≥ 20 days**<br>**rejection n=16**<br>**no rejection n=11**	rejection<br>Ln (conc) [pg/ml] mean±SD	no rejection<br>Ln (conc) [pg/ml] mean±SD	T (df)	P	Effect size<br>Cohen's d
CCL3	1.3±0.7	0.7±0.6	2.2 (25)	0.04	0.6
CCL4	4.1±0.4	3.7±0.4	2.1 (25)	0.04	0.4
INFα2	4.1±0.5	3.7±0.34	2.2 (25)	0.04	0.4
